# Over representation of the A allele in the IL23R rs1004819 polymorphism in M694V homozygote non-responsive FMF patients

**DOI:** 10.1186/1546-0096-13-S1-P87

**Published:** 2015-09-28

**Authors:** E Pras, S Dahan, A Epstein, I Ben Zvi, D Marek-Yagel, Y Shinar, M Lidar, A Livneh

**Affiliations:** 1 Sheba Medical Center, Ramat Gan, Israel; 2 Tel Aviv University, Tel Aviv, Israel

## Objectives

Recent studies have shown that interleukin-23 receptor (IL23R) polymorphisms confer susceptibility to ankylosing spondylitis, psoriasis, psoriatic arthritis and Crohn's disease. The A allele of rs1004819 was found in a significantly higher frequency among patients suffering from these diseases compared to controls. We aimed to determine the affect of rs1004819 in M694V homozygote FMF patients.

## Methods

We typed 59 M694V homozygote FMF patients for the rs1004819 polymorphism, 27 of whom were defined as non responders to colchicine treatment. In addition we typed 57 ethnically matched controls.

## Results

We found an over representation of the A allele in the non responders (A-29, G-25) as compared to the controls (A-33, G-77) (p=0.01), while a similar frequency was found between the responders (A-24, G-40) and the controls (p= 0.3).

## Conclusions

These results suggest that IL23 and its pathways are involved in the FMF inflammatory response. This association may provide new insights and treatment possibilities for FMF patients who do not respond to colchicine.

